# Effect of Electric Field Gradient on Sub-nanometer Spatial Resolution of Tip-enhanced Raman Spectroscopy

**DOI:** 10.1038/srep09240

**Published:** 2015-03-18

**Authors:** Lingyan Meng, Zhilin Yang, Jianing Chen, Mengtao Sun

**Affiliations:** 1 Beijing National Laboratory for Condensed Matter Physics, Institute of Physics, Chinese Academy of Sciences, Beijing. 100190, China; 2 Department of Physics, Xiamen University, Xiamen. 361005, China; 3 Collaborative Innovation Center of Quantum Matter, Beijing 100871, China

## Abstract

Tip-enhanced Raman spectroscopy (TERS) with sub-nanometer spatial resolution has been recently demonstrated experimentally. However, the physical mechanism underlying is still under discussion. Here we theoretically investigate the electric field gradient of a coupled tip-substrate system. Our calculations suggest that the ultra-high spatial resolution of TERS can be partially attributed to the electric field gradient effect owning to its tighter spatial confinement and sensitivity to the infrared (IR)-active of molecules. Particularly, in the case of TERS of flat-lying H_2_TBPP molecules,we find the electric field gradient enhancement is the dominating factor for the high spatial resolution, which qualitatively coincides with previous experimental report. Our theoretical study offers a new paradigm for understanding the mechanisms of the ultra-high spatial resolution demonstrated in tip-enhanced spectroscopy which is of importance but neglected.

Tip-enhanced Raman spectroscopy (TERS) is a particularly important technique for molecule analysis on the nanometer scale because of its high detection sensitivity and spatial resolution^1^,^2^,^3^,^4^,^5^,^6^,^7^. Further improving of its spatial resolution is an important step to obtain highly resolved optical images of nanometric objects^8^,^9^,^10^,^11^,^12^. In TERS the near-field electromagnetic coupling of diffraction limited incident light to a metal or metalized scanning probe microscopy (SPM) tip leads to a huge localized electromagnetic field enhancement in the narrow nanogap between tip and substrate^13^,^14^. The localized field enhancement yields an ultra-high spatial resolution breaking down the diffraction limit of light. The highest theoretically calculated spatial resolution of TERS can be 2 nm, which is closely related to the spatial extent of the localized electromagnetic field^15^. Higher order nonlinear effects are also considered to be related to such ultra-high resolution^16^. However, these physical mechanisms are not sufficient to comprehensively interpret the sub-nanometer spatial resolution in TERS. Researchers spared no effort to improve the TERS resolution experimentally^17^,^18^,^19^, and recently, a spatial resolution of 0.5 nm for TERS has been demonstrated experimentally under ultrahigh vacuum and low temperature^1^. Although there are several theories proposed to understand this high spectral spatial resolution, the physical mechanism is ongoing under discussion.

Here we show by theoretical calculations that for flat-lying H_2_TBPP molecules and H_2_TBPP molecules with a small tilted angle, the electric field gradient has a larger effect than the electric field in terms of the spectral spatial resolution. The calculated spectral spatial resolution, Raman and infrared spectra coincide with the experimental results indicating the strong influence of electric field gradient on the tip-enhanced spectroscopy^1^.

## Results

In tip-enhanced Raman spectroscopy, both Raman-active modes and infrared (IR)-active modes can be simultaneously observed *in situ*^13^,^20^,^21^. The IR-active modes are attributed to the electric field gradient effect. Particularly, for a molecule placed in an inhomogeneous electromagnetic field, the Hamiltonian for the Raman spectra can be written as^22^,
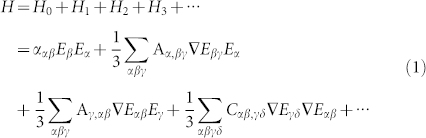
where *α_αβ_*, A*_α,βγ_* and *C_αβ,γδ_* are the electric dipole-dipole polarizability, dipole-quadrupole polarizability and quadrupole-quadrupole polarizability, respectively, describing the distortion of the molecule by an external electric field. *E_α_*, *E_β_* and *E_γ_* are the external electric field. 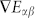, 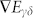 and 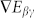 are the electric field gradient. The first term of Eq. (1) accounts for the dipole Raman, and the higher order including A*_α,βγ_* and *C_αβ,γδ_* account for the electric field gradient multiple Raman corresponding to IR-active modes^23^,^24^,^25^. The selection rules for the four terms in Eq. (1) can be obtained by the same manner in the following form^23^,
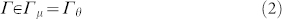
where Γ is the irreducible representation.

According to Eq. (1), the intensities of vibrational modes of a molecule can be written as
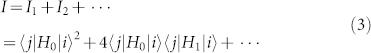
where the Raman shift is ignored, and *H*_1_ = *H*_2_ is omitted. Only the first two terms are big enough to be taken into account even in the high electric field gradient regions. Therefore, the intensity ratio of Raman-active mode to IR-active modes can be written as
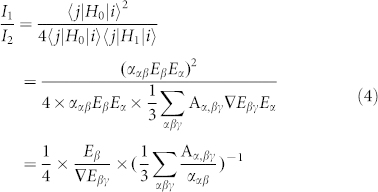
In Eq. (4), the contribution of the term
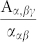
is related to polarizability of molecule, and the term 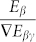
represents the plasmonic contribution from the tip. If we only consider the plasmon terms, then we obtained,
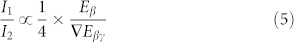
which describes the ratio of electric field enhancement to the electric field gradient enhancement.

**Figure 1:**
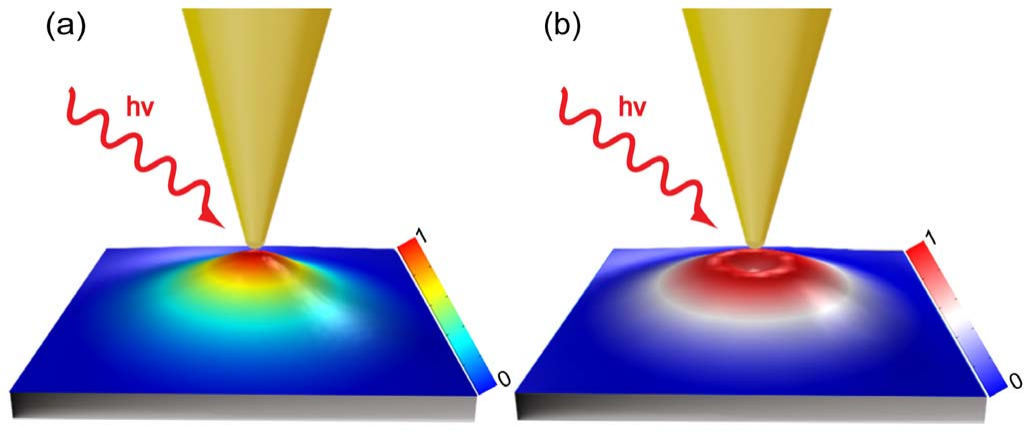
Schematics of electric field (a) and electric field gradient (b) intensity distribution of the plane between the tip and substrate in TERS configuration.

To study in detail the electric field enhancement and the electric gradient enhancement in TERS, we perform a numerical simulation, where a model of a conical gold tip on a silver substrate is employed. The exact TERS configuration is shown in Fig. 1 where a gold tip with final radius of 2 nm and a full cone angle ϕ is placed 1 nm above a silver substrate. The tip-substrate is illuminated with a p-polarized plane wave at an angle of 60° relative to the tip, and its electric field amplitude is set at 1.0 V/m.

Fig. 1(a) is the calculated intensity distribution of electric field and Fig. 1(b) is counter part of the electric field gradient where the full cone angle is 20°. Actually, the maximum of electric field enhancement is not equal to that of electric field gradient enhancement. To distinctly distinguish the spatial patterns and intensities of the two quantities, different color bars were used. We observe different spatial pattern for the two quantities. The maximum of the electric field enhancement locates at the center directly below the tip, in contrast the maximum of the electric field gradient forms a ring shape below the tip. Generally, the Raman active modes arise from the contribution of electric field while the infrared active modes are attributed to the electric field gradient effect^22^. These two fields indicate different active area for the dipole Raman and field gradient Raman.

**Figure 2:**
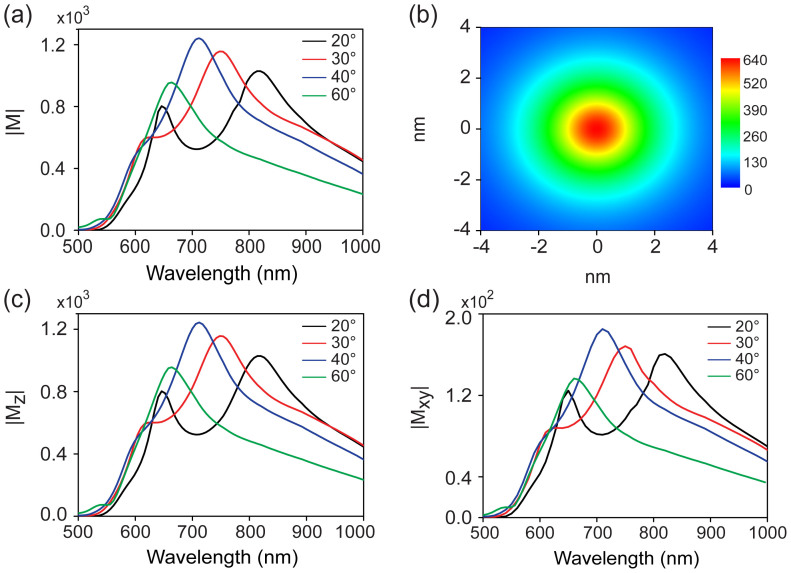
(a) The maximum total electric field enhancement (defined as |M| = |E_loc_/E_in_|) as a function of wavelength and cone angle of the tip. (b) The total electric field distribution of the plane between the tip and substrate, where ϕ = 20° and the incident wavelength is 632.8 nm. (c) The maximum vertical electric field enhancement (|M_z_|), and (d) the maximum horizontal electric field enhancement (|M_xy_|) as a function of wavelength and cone angle of the tip.

Fig. 2(a) shows the FEM simulation of the maximum total electric field enhancement (defined as the ratio between the maximum local field E_loc_ and the incident field E_in_ amplitude, |M| = |E_loc_/E_in_|) at the center of the nanogap between the tip and the substrate as a function of incident wavelength and cone angle of the tip. In all cases we observe two resonance behaviors which are attributed to the vertical (long wavelength) and the horizontal (short wavelength) dipole resonance respectively (see Fig. S1). Both the vertical and horizontal dipole resonances are red shift as decreasing the cone angle but with different speed. At ϕ = 20°, these two resonance are well separated exhibiting two discrete peaks.

We choose this tip (ϕ = 20°) and horizontal dipole resonance, which coincides with the vibration orientation of flat-lying H_2_TBPP molecules, as our research object to reveal the mechanism under the ultra-high spatial resolution of TERS. Fig. 2 (b) shows the total electric field distribution of the plane at the center of the tip and substrate with excitation wavelength of 632.8 nm. The figure clearly shows that the highest electric field enhancement is located at the center of the plane. It is believed that the spatial resolution of TERS is limited by the confinement of the electromagnetic field. Fig. 2(b) shows that this spatial extent is about 2 nm in diameter. Note that the TERS enhancement factor is proportional to the fourth power of the local electric field enhancement (|E_loc_/E_in_|^4^), which reveals an even higher spatial resolution of TERS. In Fig. 2 (c) and 2 (d), we show the dependence of the maximum vertical and horizontal electric field enhancement, (|M_z_|), and (|M_xy_|) respectively, on the wavelength and cone angle of the tip. The plasmon resonances properties for both orientations are similar to that of the total electric field. It should be noted that the maximum of the two quantities are not from the same position. The maximum horizontal electric field enhancement is located within a ring shape, while the maximum vertical electric field enhancement is located at the center of the 2D plane that can be demonstrated in the following section of the paper and the supporting information (Fig. S2(a)). As a consequence, the maximum vertical electric field enhancement can be obtained at the center of the 2D plane which is approximately equal to the total electric field enhancement (see Fig. 2 (a) and (c)), where the horizontal electric field is near to zero.

**Figure 3:**
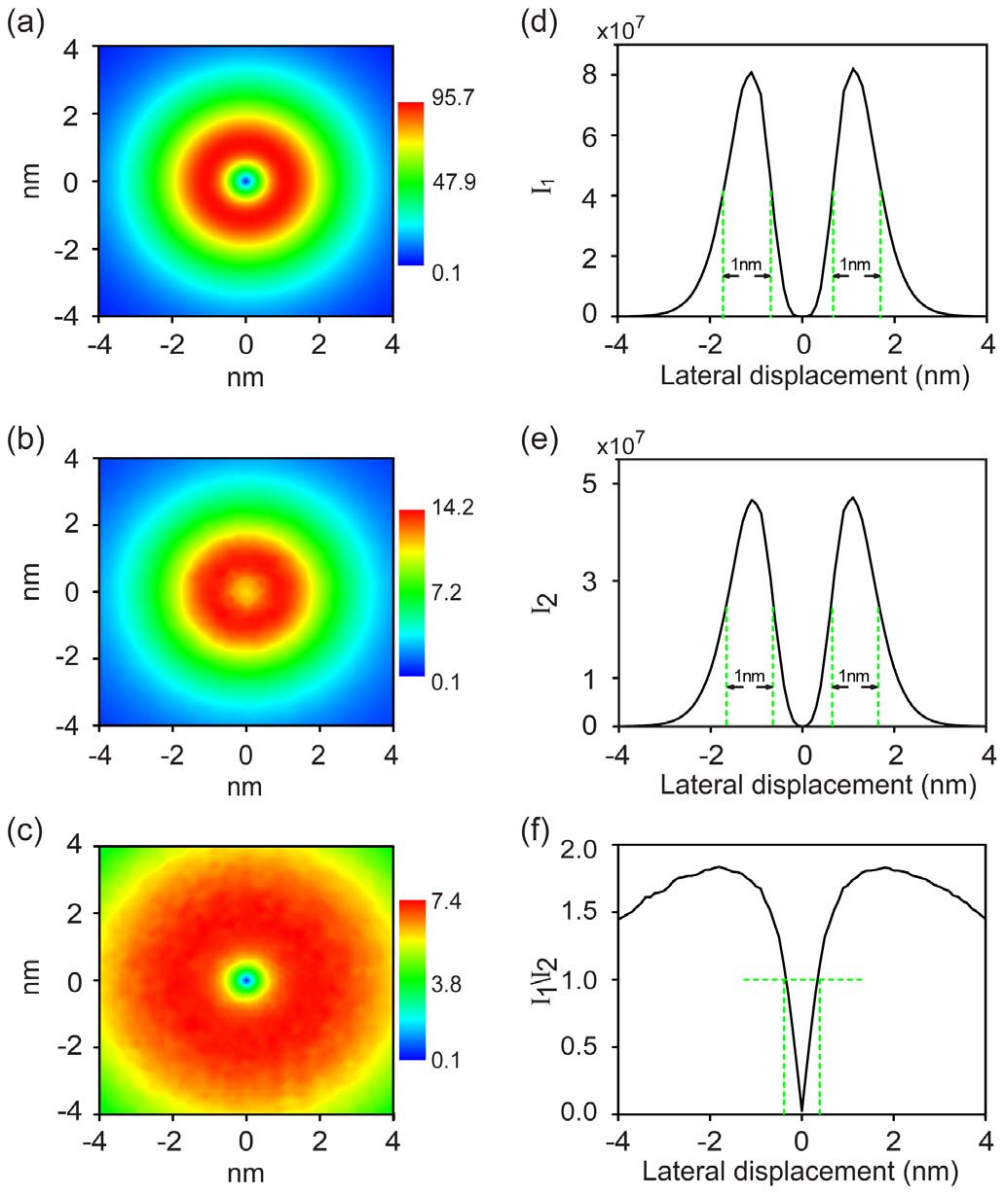
(a) The horizontal electric field and (b) horizontal electric field gradient distribution of the plane between the tip and substrate, where d = 1 nm, ϕ = 20°. (c) The ratio of (a) over (b). (d) and (e) The I_1_, I_2_ and I_1_/I_2_ is plotted as a function of the lateral displacement which can reveal the spatial resolution of I_1_, I_2_ by full width at half maximum (FWHM). (f) I_1_/I_2_ is plotted as a function of the lateral displacement which describes the ratio of electric field to its gradient. The electric gradient is in unit of au. The excitation wavelength is 632.8 nm.

In Fig. 3 we study the electric field and its gradient field distributions, and their contributions to the TERS resolution in the horizontal orientation at the wavelength 632.8 nm. As shown in Fig. 3 (a), the highest horizontal electric field enhancement (|M_xy_|) forms a ring shape with 0.7 < r < 1.7 nm (r is the distance away from the center of the plane) which has a maximum value around 1 × 10^2^. In Fig. 3 (b), the strongest electric field gradient also forms a ring shape with 0.7 < r < 1.7 nm showing the maximum of electric field gradient enhancement (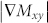) of 14.2 in atomic units. Fig. 3 (c) shows the distribution of the ratio of electric field enhancement to electric field gradient enhancement (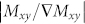). The spatial pattern forms a wider ring shape showing the maximum ratio of 7.4. This value arises from the consideration of only the second term in Equation (1). If we also consider the third term which is equal to the second term, the ratio is 3.7.

Fig. 3 (d)–(f) shows profiles of I_1_, I_2_ and I_1_/I_2_ plotted as function of the lateral displacement of the midway on the 2D plane. The spatial resolution is defined as the full width at half maximum (FWHM) in these profiles. As shown in Fig. 3d, the maximum I_1_is 8.0 × 10^7^ and the spatial resolution can be as high as 1 nm. Fig. 3e shows that the maximum I_2_ is 4.7 × 10^7^ and the spatial resolution is 1 nm. In Fig. 3f, the maximum ratio of I_1_ to I_2_ is 1.8 located at *r* = ± 1.8 nm. Importantly, the ratio I_1_/I_2_ is less than 1 at −0.4 < r < 0.4 nm, which reveals a more contribution of electric field gradient to the ultra-high spatial resolution of TERS than the electric field in the spatial extent of 0.8 nm in diameter directly below the tip, even though the same TERS resolutions of 1 nm are obtained from the two fields. If considering the combined effect of these two plasmon fields, we should obtain an even higher spatial resolution below 1 nm in TERS experiments.

**Figure 4:**
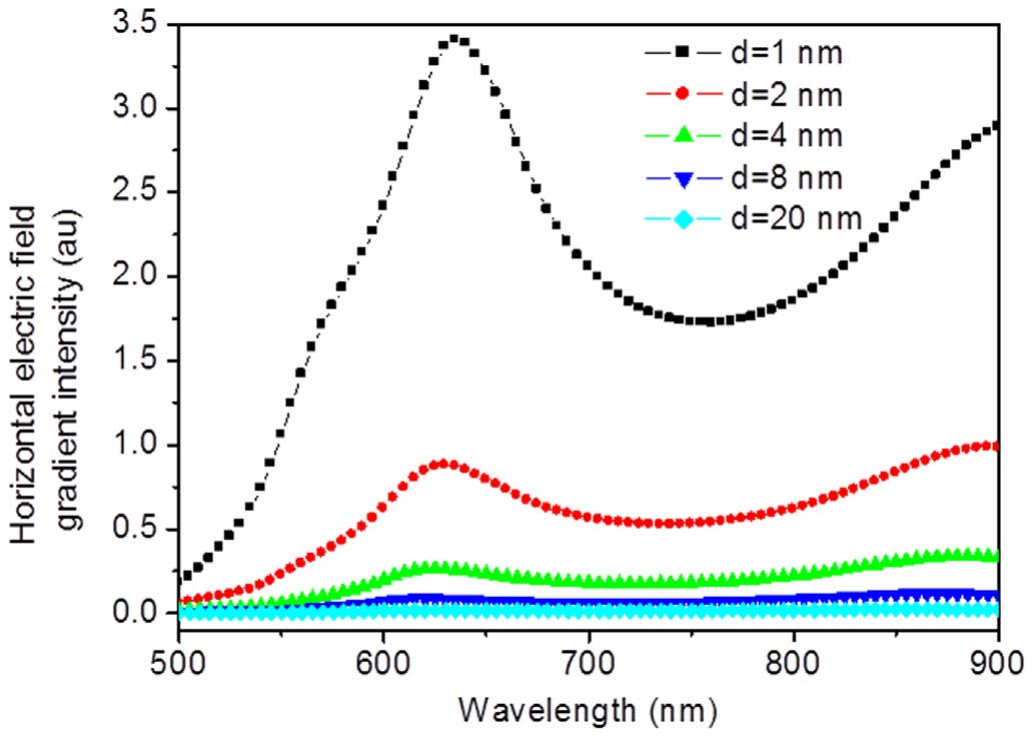
Dependence of horizontal electric field gradient intensity on the tip-substrate distance.

It is generally known that the electric field enhancement strongly depends on the tip-substrate distance in TERS system^13^,^14^. The horizontal and vertical electric field gradient are from the derivatives of the total electric field in the horizontal and vertical orientations, respectively. Hence, we can conclude that the electric field gradient effect is also strongly dependent on the distance between the tip and substrate. The calculated results of a gold tip with 20 nm radius above a silver substrate with different distances of tip-substrate can well support our conclusion (see Fig. 4).

## Discussion

It has been long believed that the strongest Raman signals of molecules can be achieved when the polarized direction of the external excitation electric field is in line with the vibration direction of the molecules. However, as these results demonstrate, the horizontal electric field gradient could have a more dominant contribution to the TERS resolution than the electric field for flat-lying H_2_TBPP molecules. Base on the electric field gradient effect, it is very helpful to understand the underlying physical mechanism of TERS, and obtain the highly resolved optical images of nanometric objects by reasonable optimal design.

**Figure 5:**
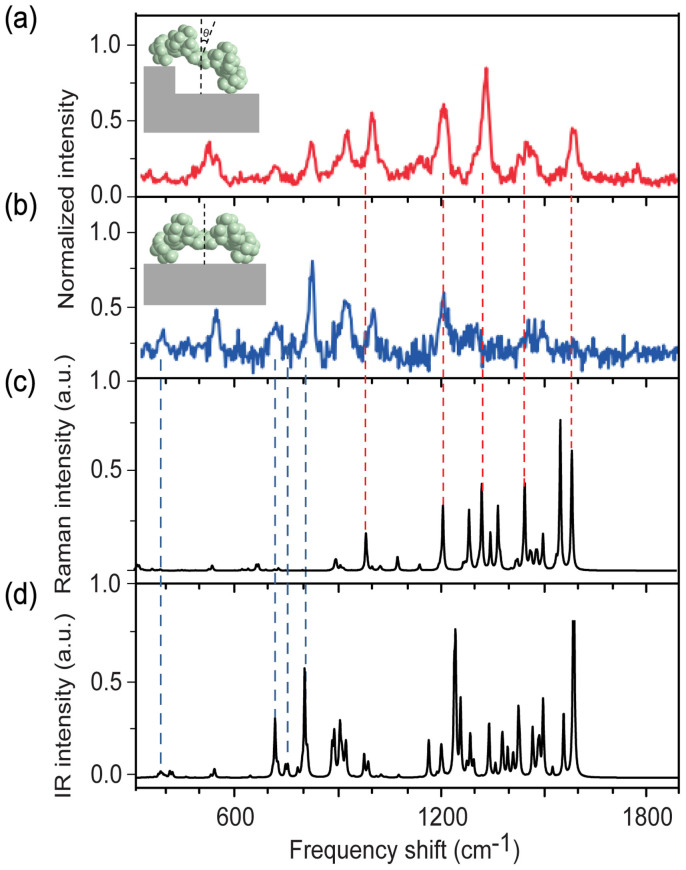
(a) and (b) Single-molecule TERS spectra for an isolated H_2_TBPP molecule adsorbed on the terrace or at the step edge. (c) and (d) Calculated TERS spectra of H_2_TBPP. The inset image in (a) shows the schematic image of tilted H_2_TBPP molecule with tilt angle of 30°. The inset image in (b) shows the schematic image of a flat-lying H2TBPP molecule. (Adapted from Ref. 1 with permission of Nature, Copyright 2013).

We provide more evidence of the important contribution of electric field gradient by showing the existence of infrared active modes of molecules in the TERS spectroscopy. Fig. 5 (a) and (b) show the previous experimental TERS spectra of H_2_TBPP molecules by Dong group from Ref.1, where the tilt angle of the molecule is 30° and 0°, respectively. Density functional theory (DFT) calculations were performed to obtain the Raman- and IR- active modes of a single H_2_TBPP molecule in free space, as shown in Fig. 5 (c) and (d). It can be seen clearly that there are four IR-active modes at the shorter frequency shift (see blue dotted vertical lines) and five Raman-active modes at the longer frequency shift (see red dotted vertical lines). As the IR-active modes are only attributed from the electric field gradient effect, these results provide a strong evidence of its important contribution to the Raman signals. Furthermore, the intensity ratio of the IR-active mode to the Raman active mode for flat-lying H_2_TBPP molecules is stronger than that for molecules perpendicularly adsorbed on the substrate. That agrees well with the variation trend in Fig. 5 (a) and (b) where as the tilt angle of the molecule varies from 0° to 30°, the relative intensity for IR-active modes decrease while for the Raman-active modes increases. This result demonstrates that the electric field gradient plays a dominant role for a horizontal orientation of molecules while the electric field is more important in the vertical orientation. By improving the electric field enhancement or electric field gradient enhancement, we can alternatively obtain the high quality Raman-active modes or IR-active modes of a molecule, even the whole vibration modes. It is quite useful for future optimal design of TERS experiment.

In our previous theoretical calculations, it has been revealed that the spatial resolutions from the contribution of horizontal electric field and electric field gradient are 1 nm for the flat-lying H_2_TBPP molecules. If considering the contribution of vertical electric field and electric field gradient, the spatial resolutions are within 2 nm (see Fig. S2). However, as shown in Fig. S1(f), the maximum ratio of I_2_/I_1 _is only 0.14 which reveals that the electric field but not the electric field gradient has a dominant contribution to the ultra-high spatial resolution of TERS in the vertical orientation. Note that the tip diameter in our calculations is set at 2 nm. If we further decrease the tip size, the spatial resolution should be higher. In fact, the size of tip apex in a real STM based TERS system is only several atoms.

In this letter, we propose a reasonable physical mechanism of ultra-high spatial resolution of TERS based on electric field gradient effect. The cone angle of the tip has a significant influence on the electric field enhancement, electric field gradient enhancement and spatial resolution. For the flat-lying H_2_TBPP molecules, the electric field gradient has a dominant contribution to the TERS resolution yielding an ultra-high spatial resolution as high as 1 nm. Our work opens up new realms for investigation of ultra-spatial resolution TERS.

## Methods

The calculations of electromagnetic field for TERS are carried out by the finite element method (FEM), which numerically solves the Maxwell's equations^26^. These simulations were performed by using COMSOL Multiphysics (version 4.3b). The optical constants for Au and Ag were taken from Ref. 27. Perfectly matched layer (PML) boundary condition and scattering boundary condition were used for all simulations. To accurately simulate the 1 nm tip–substrate distance, the mesh size was set to be 0.25 nm.

To provide more evidence of the important contribution of electric field gradient to the ultra-high spatial resolution of TERS by showing the existence of infrared active modes of molecules, density functional theory (DFT) calculations were performed to obtain the vibrational modes of a single H_2_TBPP molecule. The calculations of vibrational spectra of H_2_TBPP were carried out, using the Gaussian 09 software, the B3LYP functional and 6–31G(d) basis set.

## Author Contributions

J.C., Z.Y. and M.S. supervised the project. M.S., Z.Y. and J.C. proposed the idea. L.M. and M.S. performed the calculations. M.S., L.M., Z.Y. and J.C. analyzed data. M.S. and L.M. wrote the paper. All authors were involved in the discussion and revision of the manuscript.

## Supplementary Material

Supplementary Information supporting information
