# Supplementary material for: Effect of Electric Field Gradient on Sub-nanometer Spatial Resolution of Tip-enhanced Raman Spectroscopy
Source: Sci Rep. 2015 Mar 18;5:9240. doi: 10.1038/srep09240 (PMC4363867; doi:10.1038/srep09240)
Supplement: Supplementary Information — supporting information [file srep09240-s1.pdf]

Effect of Electric Field Gradient on Sub-nanometer Spatial Resolution of Tip-enhanced Raman Spectroscopy

Lingyan Meng,[1,2] Zhilin Yang,[2,*] Jianing Chen,[1,3,*] Mengtao Sun[1,*]

[1]*Beijing National Laboratory for Condensed Matter Physics, Institute of Physics, Chinese Academy of Sciences, Beijing, 100190, China*

[2]*Department of Physics, Xiamen University, Xiamen, 361005, China*

[3] *Collaborative Innovation Center of Quantum Matter, Beijing 100871, China*

* Corresponding authors. E-mail: mtsun@iphy.ac.cn (M. T. Sun), jnchen@iphy.ac.cn (J. N. Chen) or zlyang@xmu.edu.cn (Z. L. Yang).

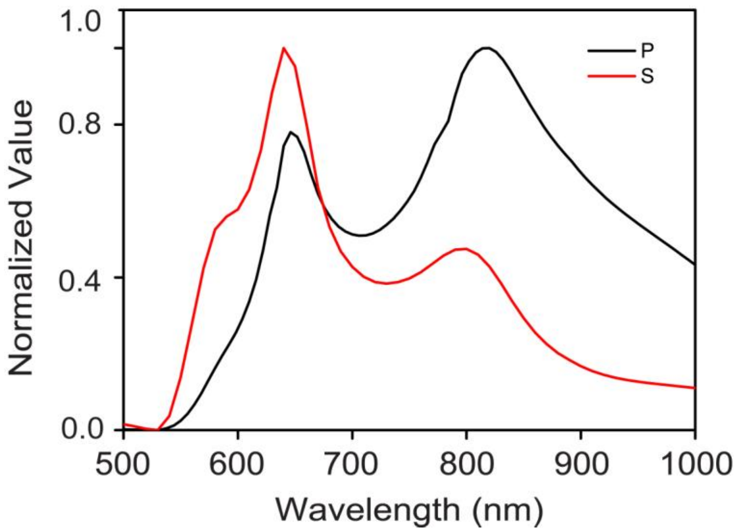

Figure S1 | Normalized total electric field enhancement (|M|) as a function of wavelength and polarization orientation, where d=1 nm, ϕ=20°.

FIG.S2 shows the dependence of electric field enhancement on the wavelength and the polarization orientation. Note that the intensities were normalized. For the P-polarization, the relative intensity of resonance peak at longer wavelength is higher than that at the shorter wavelength due to the strong coupling effect between the tip and substrate. While we change the polarization orientation to S-polarization, the resonance peak at longer wavelength is suppressed leading to a lower relative intensity than that of resonance peak at shorter wavelength. Therefore, two resonance peaks are attributed to the vertical (long wavelength) and the horizontal (short wavelength) dipole resonance, respectively.

When the molecules perpendicular adsorbed on the substrate, the vertical electric field and its gradient along the tip axis play the most important role. In Fig.S1 we study the vertical electric field and its gradient field distributions, and their contributions to the TERS resolution in the horizontal orientation at the wavelength 632.8 nm. Fig. S1(a) shows the highest electric field enhancement ($|M_z|$) occurs at the center of the 2D plane showing the maximum field enhancement of 636. As shown in Fig. S1(b) the strongest electric field gradient ($|\nabla M_z|$) is distributed within the ring band 0.7<r<1.7 nm which is the same as that in the horizontal orientation. This can be inferred by the Laplace's equation,

$$\frac{\partial^2 E}{\partial x^2}+\frac{\partial^2 E}{\partial y^2}+\frac{\partial^2 E}{\partial z^2}=0 \qquad (5)$$

Fig. S1(c) shows the distribution of the ratio of electric field gradient enhancement over electric field enhancement ($|\nabla M_z / M_z|$) where the maximum ratio is only 0.036. Fig. S1(d)-(f) shows profiles of I_1, I_2 and I_1/I_2 plotted as function of the lateral displacement of the midway on the 2D plane. As shown in Fig.S1(d), the maximum I_1 is $1.6\text{x}10^{11}$ and the spatial resolution is 1.5 nm. Fig. S1(e) shows that the maximum I_2 is $1.2\text{x}10^{10}$ and the spatial resolution 2 nm. In Fig. S1(f), the maximum ratio of $I_2/I_1 \approx 0.14$ which reveals a minor contribution of electric field gradient to the ultra-high spatial resolution of TERS.

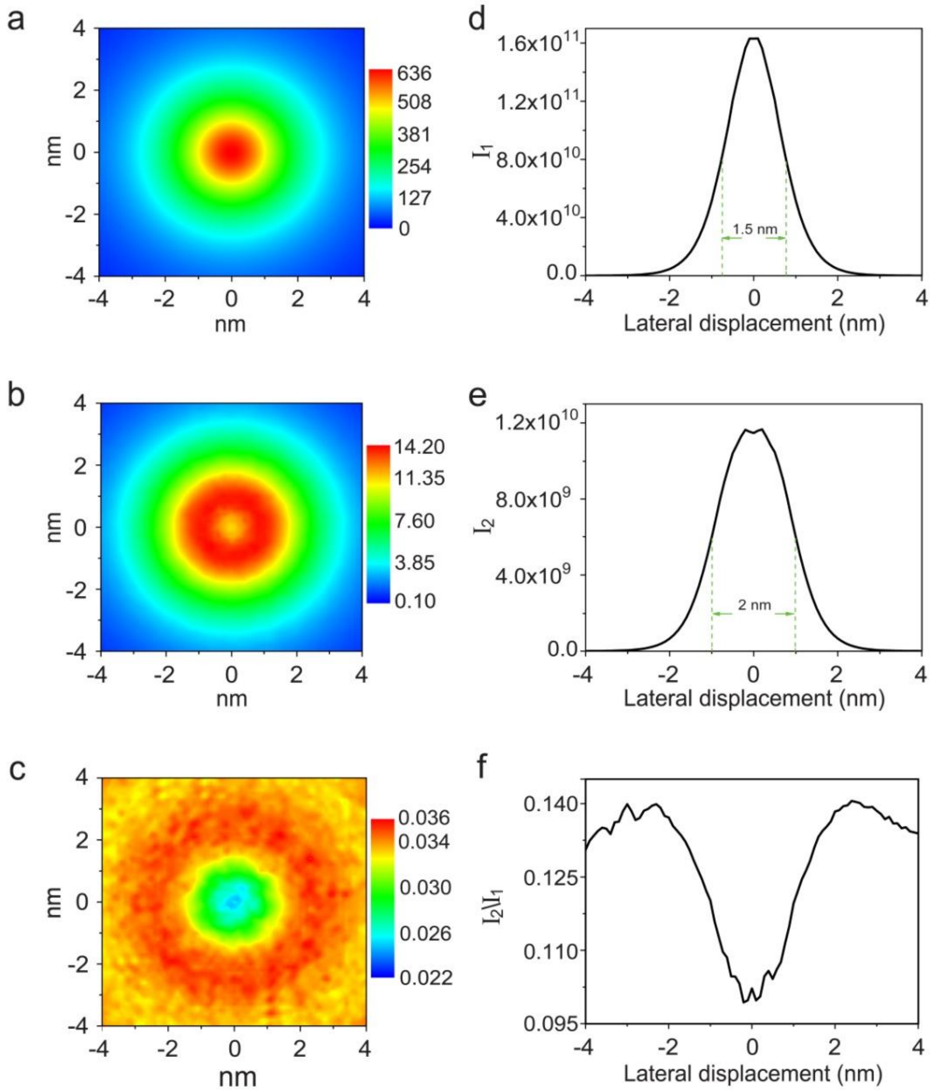

Figure S2 | (a) The vertical electric field and (b) vertical electric field gradient distribution of the plane between the tip and substrate, where d=1 nm, ϕ=20°. (c) The ratio of (b) over (a). (d) and (e) The I_1, I_2 and I_2/I_1 is plotted as a function of the lateral displacement which can reveal the spatial resolution of I_1, I_2 by full width at half maximum (FWHM). (f) I_2/I_1 is plotted as a function of the lateral displacement which describes the ratio of electric field to its gradient. The electric gradient is in unit of au which is atomic unit. The excitation wavelength is 632.8 nm.
